# Letter to the Editor: Building on Promising Evidence for Dual‐Chamber ICDs in LBBAP

**DOI:** 10.1111/anec.70118

**Published:** 2025-10-21

**Authors:** Mohammad Tayyab Qayyum

**Affiliations:** ^1^ Nishtar Medical University Multan Pakistan

Dear Editor,

We read with great interest the systematic review by Ahmad et al. on dual‐chamber implantable cardioverter defibrillators (ICDs) for left bundle branch area pacing (LBBAP) [1]. The authors commendably synthesize nascent evidence for this innovative strategy. Their findings—significant QRS narrowing (170 ± 17.4 ms to 121 ± 17.3 ms), improved LVEF (50% improvement in 2 studies), and no short‐term complications in 34 patients—are promising, suggesting LBBAP‐ICD as a viable alternative to cardiac resynchronization therapy defibrillators (CRT‐D).

Advantages like avoiding coronary sinus leads, reduced fluoroscopy, potential cost savings, and stable sensing [2] are compelling. However, as noted, significant limitations necessitate cautious interpretation. We wish to emphasize key points for future research:

1. *Need for larger RCTs*: The review is based on three small, uncontrolled cohorts (*n* = 34) [2, 3, 4]. Larger, multicenter RCTs comparing LBBAP‐ICD directly with CRT‐D are essential to establish comparative efficacy, safety, and cost‐effectiveness.
2. *Long‐term data*: With a maximum 12‐month follow‐up, long‐term lead stability, defibrillation performance, sustained LVEF improvement, and potential late complications (e.g., septal perforation) remain unknown. Extended follow‐up (≥ 3–5 years) is critical.
3. *Generalizability*: The small, selected populations (predominantly male, ~61 years) limit applicability. Future studies must include more diverse cohorts (women, older patients, various cardiomyopathies) to define true patient selection criteria.
4. *Standardization and learning curve*: Procedural standardization is still evolving. Optimal techniques and the impact of the operator learning curve on success and complications need assessment in larger studies.
5. *Formal cost‐effectiveness*: Suggested cost savings require formal economic evaluation within comparative trials.

In conclusion, Ahmad et al. provide a valuable synthesis confirming the *feasibility* and *short‐term promise* of LBBAP‐ICD. However, this review powerfully highlights the preliminary nature of the evidence. Substantial investment in robust, comparative, long‐term trials is now needed to determine if this approach fulfills its promise as a safe and effective alternative for patients.

## Author Contributions

The author takes full responsibility for the conception, design, data interpretation, and drafting of this manuscript.

## Conflicts of Interest

The author declares no conflicts of interest.

## Data Availability

The data that support the findings of this study are available on request from the corresponding author. The data are not publicly available due to privacy or ethical restrictions.
